# Therapeutic potential of cannabidiol supplementation in mitigating lipid precursors of inflammation in hepatic steatosis progression

**DOI:** 10.1186/s42238-026-00413-z

**Published:** 2026-02-25

**Authors:** Karolina Konstantynowicz-Nowicka, Mateusz Zwierz, Piotr Franciszek Kurzyna, Magdalena Zabielska-Kaczorowska, Klaudia Sztolsztener, Adrian Chabowski, Ewa Harasim-Symbor

**Affiliations:** 1 Department of Physiology, Medical University of Bialystok, Mickiewicz Str. 2C, Bialystok, 15-222 Poland; 2 Department of Physiology, Medical University of Gdańsk, Dębinki Str. 1, Gdańsk, 80-211 Poland

**Keywords:** Steatosis, High-fat diet, Inflammation, Arachidonic acid, Cannabidiol

## Abstract

**Background:**

This study investigated the effects of cannabidiol (CBD) on early-stage inflammation, a key factor in the progression of liver diseases from metabolic dysfunction-associated steatotic liver disease (MASLD) to metabolic dysfunction-associated steatohepatitis (MASH) and irreversible cirrhosis. The study focused on CBD’s influence on the pro-inflammatory n-6 and anti-inflammatory n-3 pathways, on arachidonic acid (AA) levels as an early marker of inflammation, and the expression of enzymes involved in AA metabolism, as well as inflammatory cytokines and chemokines.

**Methods:**

Forty male Wistar rats were randomly divided into four groups: control (C)—fed a standard diet and treated with cannabidiol vehicle for the last 14 days, control + cannabidiol (C + CBD) – fed a standard diet and treated with CBD for the last 14 days, high-fat diet (HFD) – fed a high-fat diet and treated with cannabidiol vehicle for the last 14 days, high-fat diet + cannabidiol (HFD + CBD)—fed a high-fat diet and treated with cannabidiol for the last 14 days. At the end of the treatment period, all the rats were fasted for 24 h, anesthetized, and sacrificed. Gas–liquid chromatography was used to measure n-6 and n-3 pathway polyunsaturated fatty acids (PUFAs) activities and AA levels in lipid fractions in the liver. The Multiplex immunoassay assessed cytokine and chemokine content in liver tissue, while Western Blot analyzed the expression of selected enzymes.

**Results:**

Initial findings indicated CBD’s potential in reducing inflammation and its therapeutic efficacy in preventing MASH development induced by HFD. The results indicated that supplementing with CBD led to a decrease in the n-6 PUFA pathway, known for its pro-inflammatory effects, and an increase in the anti-inflammatory n-3 PUFA pathway. These changes were simultaneous with lower levels of arachidonic acid, which is crucial for the formation of inflammatory mediators. CBD influenced the expression of enzymes like COX-1 and COX-2 involved in AA metabolism and reduced the levels of pro-inflammatory cytokines.

**Conclusions:**

Our observations confirmed that CBD, which affects early indicators of inflammation, has the potential to become a new and safe, promising supportive drug for hepatic inflammation and steatosis treatment.

**Graphical Abstract:**

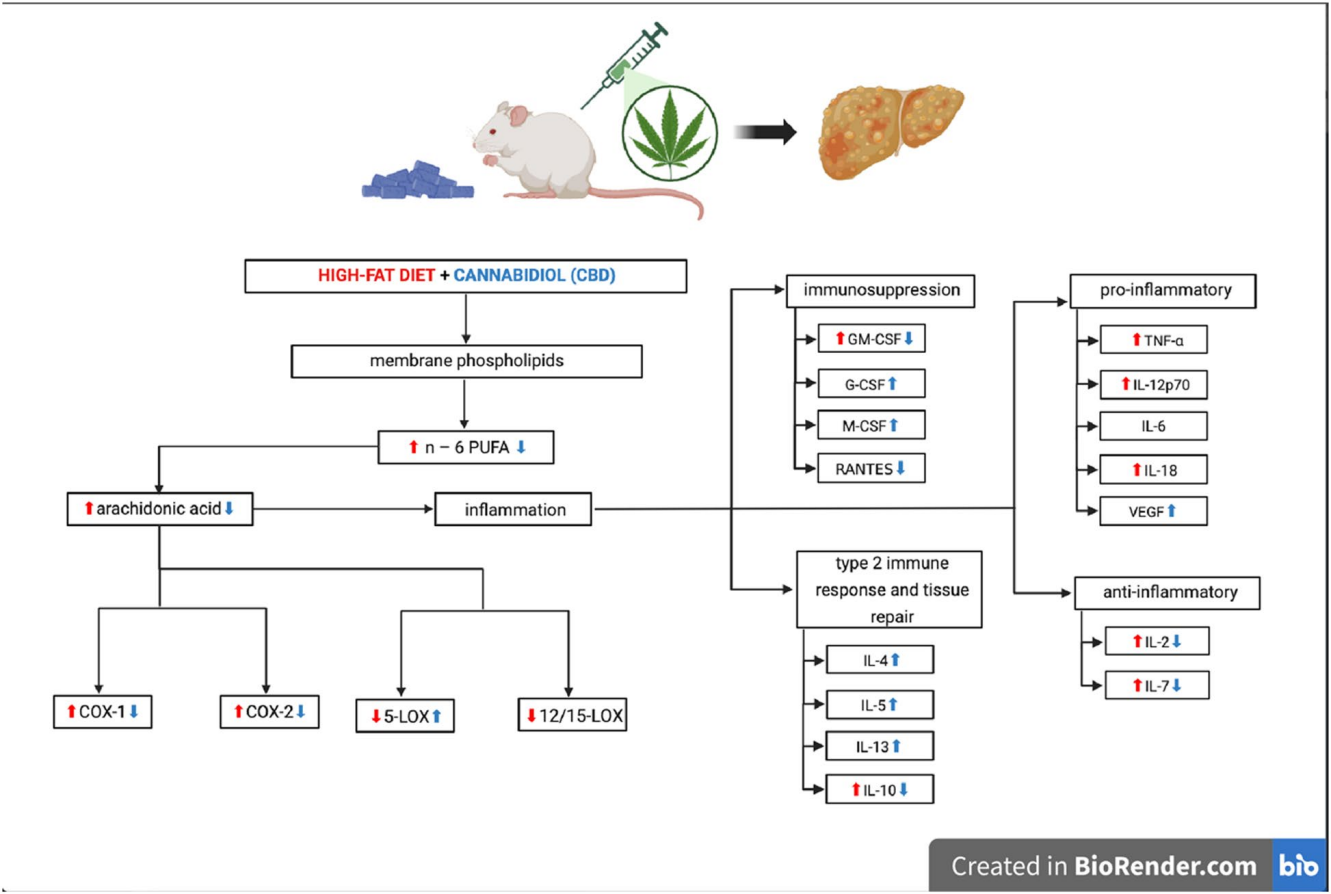

**Supplementary Information:**

The online version contains supplementary material available at 10.1186/s42238-026-00413-z.

## Introduction

Cannabidiol (CBD) is the main active ingredient in cannabis (*Cannabis sativa L*) without psychoactive properties. According to the literature, CBD demonstrates multiple beneficial effects, including neuroprotective, anti-epileptic, analgesic, and anti-tumor properties (Peng et al. 2022). However, CBD is most known for its therapeutic effects on inflammation and oxidative stress-related diseases (Atalay et al. 2019). The mechanisms of CBD’s anti-inflammatory and antioxidant effects involve its interaction with not only nuclear receptors (PPAR) or transient receptor potential (TRP) channels, but also cyclooxygenases (COX-1 and COX-2) (Sztolsztener et al. 2023; Julien et al. 2005). It seems to be even more important as COX-1 and COX-2 catalyze the creation of eicosanoids, which start with the liberation of arachidonic acid from its bound state in the cell membranes. Some studies suggest that cannabidiol also inhibits the release of this fatty acid, indicating CBD’s therapeutic potential also in liver disease treatment (Burstein 2015).

The most common liver disease, which may be caused by obesity, is metabolic dysfunction-associated steatotic liver disease (MASLD), previously known as nonalcoholic fatty liver disease (NAFLD). The initial stage in the development of MASLD is increased lipid accumulation in hepatocytes exceeding 5% of the cell volume. This excessive deposition of lipids in the liver triggers inflammation primarily through lipotoxicity. It is a process where mainly bioactive lipid fractions, like diacylglycerols (DAG), overwhelm the liver’s storage capacity, interact with many signaling pathways, disrupt organelle function, causing endoplasmic reticulum stress and mitochondrial dysfunction. Additionally, excess lipids can undergo oxidation, producing reactive oxygen species (ROS) and lipid peroxides, which cause direct cellular damage (Svegliati-Baroni et al. 2019; Sonnweber et al. 2018; Mello et al. 2016). This damage initiates an immune response associated with excessive activation of enzymes like COX-1 and COX-2, which activate the arachidonic acid pathway cascade (Sonnweber et al. 2018; Cusi 2012). According to the literature, this pathway is an early indicator of liver inflammation and is thought to be a precursor of pro-inflammatory compounds, such as eicosanoids (Sonnweber et al. 2018; Sztolsztener et al. 2020; Patterson et al. 2012). The mechanism described above shows that simple fatty liver (MASLD/NAFLD) may progress to a more severe inflammatory state (metabolic dysfunction-associated steatohepatitis—MASH/nonalcoholic steatohepatitis—NASH), which both may predispose to the development of hepatocellular carcinoma (HCC) (Khare et al. 2025). Although the pathophysiological mechanism of MASLD development and progression is known, there are no effective and safe treatment methods. Thus, looking for therapeutic agents that have high anti-inflammatory and antioxidant activities, which have a potentially protective effect on inhibiting the progression of MASLD to MASH, is of high priority. A promising therapeutic strategy that may regulate the metabolic homeostatic processes is the group of phytocannabinoids, CBD in particular (Echeverría et al. 2016; O’Sullivan 2013).

This study determined how the administration of cannabidiol to rats with a high-fat diet (HFD)-induced MASLD can prevent its development to MASH. Thus, the study focused on evaluating the effect of cannabidiol on the activity of polyunsaturated fatty acids (PUFAs) pathways, i.e., pro-inflammatory n-6 and anti-inflammatory n-3. Moreover, the study aimed to evaluate the concentration of arachidonic acid (AA; C20:4n-6) as an early indicator of inflammation in three selected lipid fractions, i.e., triacylglycerols (TAG), DAG, and free fatty acids (FFA) in an HFD-induced MASLD. This animal model was selected because it best represents human individuals who develop MASLD during obesity. The next step was to test the effect of CBD on regulating the expression of enzymes from the arachidonic acid metabolism pathway. Furthermore, the effect of CBD on the levels of inflammatory cytokines and chemokines was also evaluated.

## Materials and methods

### Chemicals and Kits

Cannabidiol with purity ≥ 99% was purchased from THC Pharm GmbH (Frankfurt, Germany), and CBD solvent containing ethanol, Tween-80, and 0.9% NaCl was from Sigma-Aldrich (Merck, Darmstadt, Germany). Standard chemicals used in chromatography, such as methanol, chloroform, hexane, and 14% boron trifluoride, were also purchased from Sigma-Aldrich (Merck, Darmstadt, Germany). The internal standard—heptadecanoic acid (C17:0), and standards of fatty acid were from Larodan Research grade lipids (Stockholm, Sweden), whereas chromatographic plates coated with silica gel (Silica Plate 60, 0.25 mm) were from Merck (Darmstadt, Germany). Chemicals used in the Western blot technique, namely radioimmunoprecipitation assay (RIPA) buffer, bicinchoninic acid (BCA), 5% BSA-containing tris-buffered saline buffer, and Tween-20 (TBST) were purchased from Sigma-Aldrich (Merck, Darmstadt, Germany). The Criterion TGX StainFree Precast Gels, Laemmli buffer, and Clarity Western ECL Substrate were purchased from Bio-Rad (Hercules, CA, USA), whereas antibodies were from various companies namely: 5-LOX from Abcam (Cambridge, UK), 12/15- LOX, COX-1, COX-2 from Santa Cruz Biotechnology (Inc., Dallas, TX, USA), and pNRF2 (Ser40) from Invitrogen Biotechnology (Waltham, MA, USA). The multiplex assay was conducted with the use of Bio-Plex Immuno-assay Kit (BioPlex Pro Rat Cytokine 23-Plex Assay) and Bio-Plex Cell Lysis Kit, both purchased from Bio-Rad (Warsaw, Poland). The ALT ELISA kit was purchased from Biorbyt (Cambridge, UK). The standard chemicals used in histological studies, such as 10% buffered formalin, hematoxylin, eosin, and 50% ethanol, were purchased from Merck (Darmstadt, Germany).

### Rat diet constituents

Standard rodent chow energy density was 2.74 kcal/g and contained: 67 kcal% carbohydrates, 25 kcal% protein, and 8 kcal% fat (Labofeed B standard, Kcynia, Poland). The selection and composition of the diet were based on literature and are available at (Nowacki et al. 2017). A high-fat diet energy density was 5.24 kcal/g and contained: 60 kcal% fat, 20 kcal% carbohydrate, and 20 kcal% protein (Research Diet, New Brunswick, NY, USA; cat No. D12492). The selection and composition of the diet were based on literature and are available at (Zalewska et al. 2019) and manufacturer protocols.

### Animals and experimental protocol

In the experiment, MASLD was induced by high-fat diet feeding for 7 weeks in male Wistar rats initially weighing 70–100 g. All the animals were housed in typical animal laboratory conditions for the duration of the investigation, which included plastic autoclavable cages, an ambient temperature of 22 ± 2 °C, a 12-h light/dark cycle, unlimited access to water, and selected chow. After a week of acclimatization and handling, 40 rats were divided randomly into four groups of ten rodents each. Animals from Group 1—control (C) and Group 2—control + cannabidiol (C + CBD) received standard rodent chow for 7 weeks, while those from Group 3—high-fat diet (HFD) and Group 4—high-fat diet + cannabidiol (HFD + CBD) were fed a high-fat diet for 7 weeks. During the last 14 days of the experiment, rats from Group 2 (C + CBD) and Group 4 (HFD + CBD) obtained intraperitoneal CBD injections at a dose of 10 mg/kg of body weight, one time a day, together with a selected diet (standard or high-fat, respectively). The dose of CBD injected into animals was based on the previous literature (Navarrete et al. 2018; Xu et al. 2019a). The CBD was dissolved in a solvent that contained ethanol, Tween-80, and 0.9% NaCl in a ratio of 3:1:16. Rats from Group 1 (C), as well as Group 3 (HFD), were also injected with CBD solvent. All along the study, the body mass of the rats was recorded. At the end of the study, 24 h after the last CBD dosage, the rats were anesthetized using an isoflurane inhalation. During the operation, the liver tissue was removed from dormant animals and frozen in liquid nitrogen. The obtained material was kept at −80^◦^C until all the measurements were conducted. The experiment was approved by the Ethical Committee for Animal Testing in Olsztyn, Poland (Approval No. 71/2018).

### Analysis of liver lipid concentrations

Lipids were extracted from the liver tissue samples using the solution of methanol: chloroform in a ratio of 1:2 as described by Folch et al. (Folch et al. 1957). The tubes containing the extracted samples were supplemented with an internal standard that contained heptadecanoic acid (C17:0). Then, the extracts were transferred onto glass chromatographic plates coated with silica gel (Silica Plate 60, 0.25 mm) for the thin-layer chromatography (TLC) technique. The identification of selected lipid fractions on dried silica plates was conducted under ultraviolet light according to two standard lines. Next, bands of separated specific lipid fractions were scraped and eluted in an appropriate buffer. Acquired lipid fractions eluents were dissolved in hexane after being transmethylated in a solution containing 14% boron trifluoride and methanol. The fatty acid methyl esters in particular lipid fractions were measured using gas–liquid chromatography (GLC, Hewlett-Packard 5890 Series II Gas Chromatograph, Agilent Technologies, Santa Clara, CA, USA) fitted with a capillary column (Hewlett-Packard-INNOWax) and an ionization detector. The amount of AA contained in the specific lipid fractions (FFA, DAG, TAG, and PH) was quantified in accordance with the retention times of standards prepared from commercially available standards of fatty acid and was expressed in nanomoles per gram of tissue. Furthermore, the activity of the n–3 and n–6 pathways was computed depending on the concentration of each particular fatty acid in the chosen fraction.

### Analysis of liver protein expression

To evaluate protein expression, a standard Western blotting technique was used, as it was previously outlined (Konstantynowicz-Nowicka et al. 2015). Protease and phosphatase inhibitors were added to the liver, and homogenized in RIPA buffer at 4 °C before the immunoblotting process. Then, using the BCA assay, the total protein content was assessed. The same protein volume (30 μg) was loaded onto Criterion TGX StainFree Precast Gels following the dilution of tissue homogenates in Laemmli buffer. After being separated by electrophoresis, all of the proteins were transferred onto nitrocellulose membranes. Following this, the membranes were blocked with 5% non-fat dried milk or 5% BSA-containing tris-buffered saline buffer including Tween-20 (TBST), and then overnight immunoblotting was conducted using the appropriate primary antibodies: 5-LOX (1:1500), 12/15- LOX, COX-1, COX-2 (1:500), and pNRF2 (Ser40) (1:1000). The appropriate horseradish peroxidase (HRP)-conjugated antibodies were then incubated with nitrocellulose membranes. The protein bands were observed using the chemiluminescence substrate (Clarity Western ECL Substrate), and the immunoblotting signals were densitometrically analyzed using the visualization system (Chemi-Doc, Image Laboratory Software; Bio-Rad, Warsaw, Poland). The obtained protein of interest and the image of the total protein visible in the membrane upon transfer overlapped in the ImageLab system to perform the standardization, with the control group expressed as 100%.

### Analysis of liver cytokines and chemokines concentrations

All of the liver lysates were prepared with the use of Bio-Plex Cell Lysis Kit following the manufacturer’s protocol before the multiplex assay method. At the beginning, the samples of liver tissue were rinsed in ice-cold wash buffer and immediately transferred to the tissue grinder with lysing solution containing PMSF and two lysing factors provided by the manufacturer. Next, the tissue was ground on ice, transferred to a clean tube, and frozen at −80◦C. After thawing, the samples were briefly sonicated, centrifuged (at 15,000 xg for 10 min at 4^◦^C), and the supernatant was collected. Following a centrifugation, the total protein concentration was measured, and to obtain the total protein concentration range 200–900 μg/ml, the supernatant was diluted 1:4 with the Dilution Buffer contained in the kit. Subsequently, the supernatants were kept at −80 °C until they were analyzed. Cytokines and chemokines; interferon (IFN)-γ, vascular endothelial growth factor (VEGF), tumor necrosis factor (TNF)-α, interleukin (IL)- 1α, IL-1β, IL-2, IL-4, IL-5, IL-6, IL-7, IL-10, IL-12p70, IL-13, IL-17A, IL-18, macrophage inflammatory protein (MIP)−3α, granulocyte colony stimulating factor (G-CSF), granulocyte macrophage colony stimulating factor (GM-CSF), growth-regulated oncogenes/keratinocyte chemoattractant (GRO/KC), macrophage colony-stimulating factor (M-CSF), macrophage inflammatory protein (MIP)−1α, monocyte chemoattractant protein (MCP)−1, and regulated on activation, normal T-cell expressed and secreted (RANTES) concentrations were determined by the multiplex assay (Bio-Plex Immuno- assay Kit, BioPlex Pro Rat Cytokine 23-Plex Assay).

The 96-well assay plate was first filled with diluted magnetic beads, and then it was twice washed with Bio-Plex Wash Buffer. Samples and blank standards were then added to specific wells, and the plates were shaken for one hour. Following a sequence of successive washes, each well was treated with the detection antibodies, which were then incubated for 30 min. Following three plate washes, each well on the plate was filled with streptavidin–phycoerythrin (SA-PE) solution, and the plate was incubated for 10 min. Next, the beads were resuspended in the assay buffer and shaken for 30 s. Then, the Bio-Plex 200 system (Bio-Rad Laboratories, Inc.; Hercules, CA, USA) linked to Bio-Plex Manager Software was used to read the 96-well plate. The concentrations of cytokines and chemokines were evaluated in relation to the individual standard curves established for each parameter. The data were expressed as picograms of protein per milliliter.

### Enzyme-linked immunosorbent assay

A commercially available enzyme-linked immunosorbent assay kit was used to measure the concentration of the alanine aminotransferase (ALT) in samples of plasma. The assay was conducted according to the manufacturer’s protocol, and before the procedure, the samples of plasma were diluted twofold. The absorbance was measured spectrophotometrically at a wavelength of 450 nm in a Synergy H1TM microplate reader (BioTek Instruments, Winooski, VT, USA), and the calculated ALT concentrations were based on the obtained standard curve and expressed in nanograms per milliliter of plasma.

### Histopathological examination

The liver samples of the same fragment of the lobe from each rat were collected for histological studies. They were fixed in 10% buffered formalin and processed routinely for embedding in paraffin. After cutting the sections into pieces 4 µm in thickness, they were stained with hematoxylin–eosin. Then, the sections were briefly rinsed in 50% ethanol, followed by rinsing in tap water, and stained with Mayer’s hematoxylin for 2 min. The sections were rinsed in tap water by customary procedure and mounted in glycerin jelly. The results of staining were evaluated by two experienced, independent histologists in an Olympus BX41 microscope with an Olympus DP12 camera under a magnification of 200x (20 × lens and 10 × eyepiece) with Columbus software.

### Statistical analysis

The data are presented as mean values ± standard deviation (SD) based on ten (GLC), eight (Bioplex), nine (ELISA), or six (WB, histology) independent determinations. Bartlett’s test and the Shapiro–Wilk test were used to verify the homogeneity of the variance and the normality of the data distribution. Using GraphPad Prism 8.2.1 (GraphPad Software, San Diego, CA, USA), the statistical evaluation was carried out by two-way ANOVA and the corresponding post-hoc test (pairwise Student’s t-test). At *p* < 0.05, the differences were considered statistically significant.

## Results

### The concentration of AA in selected lipid fractions

**Fig. 1:**
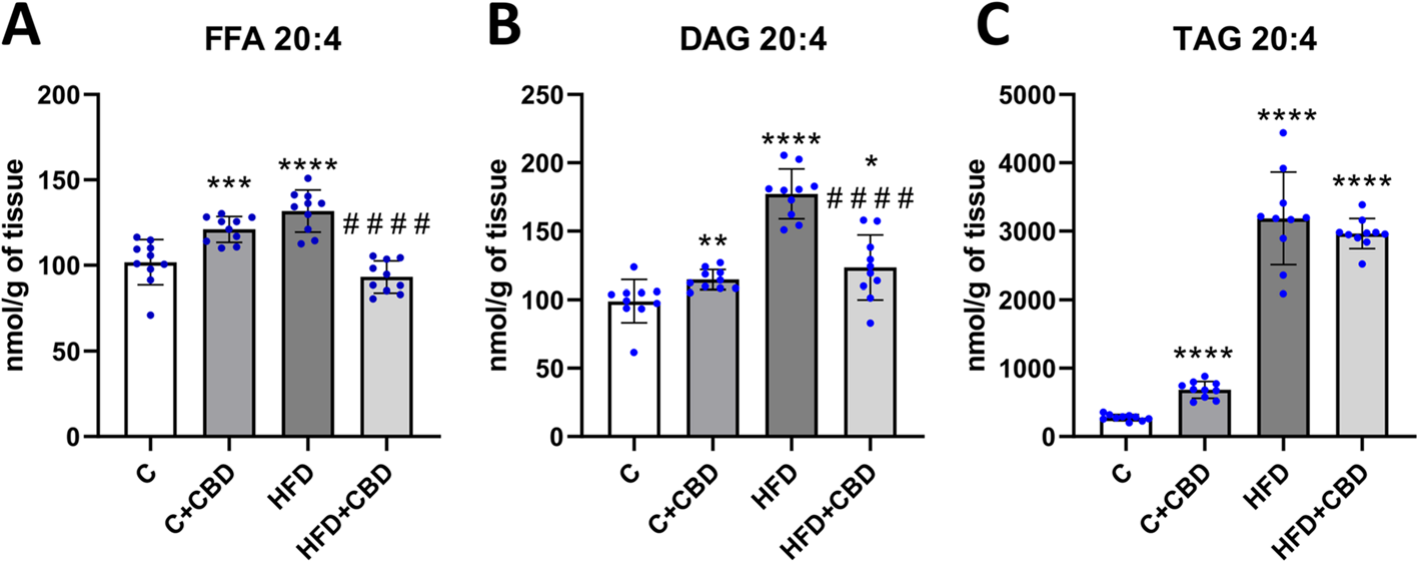
The arachidonic acid (AA, 20:4) concentration. The concentration of arachidonic acid (AA, 20:4) in (**A**) the FFA-free fatty acid; (**B**) DAG – diacylglycerol; (**C**) TAG – triacylglycerol lipid fractions in the liver tissue after intraperitoneal injections of CBD during the last 2 weeks of the experiment to rats fed a standard diet (**C**) or high-fat diet (HFD) for 7 weeks. AA concentration was measured by the gas–liquid chromatography (GLC) method. The results are expressed as ten independent determinations in each experimental group with mean values ± SD. *–p < 0.05; **–p < 0.01; ***–p < 0.001; ****–p < *0.0001* significant change vs control group; *#–p* < *0.05; ##–p* < *0.01; ###–p* < *0.001; ####–p* < *0.0001* significant change vs HFD group

A significant increase in the AA level in the FFA fraction in the C + CBD group and HFD group compared to the control group was observed (C + CBD: + 18.89%, *p* < *0.001*, HFD: 28.89%, *p* < *0.0001*, Fig. 1A). Additionally, there was a substantial decrease in the HFD + CBD group in comparison with the rats fed with HFD (HFD + CBD: −28.98%, *p* < *0.0001*, Fig. 1A). In DAG fraction, the AA level was markedly enhanced in all the groups compared to the control group (C + CBD: + 15.98%, *p* < *0.01*, HFD: + 79.18%, *p* < *0.0001*, HFD + CBD: + 25.70%, *p* < *0.05*, Fig. 1B), while a noteworthy decline was noticed in rats treated simultaneously with HFD and CBD in comparison with the rats fed solely with HFD (HFD + CBD: −29.84%, *p* < *0.0001*, Fig. 1B). It was also detected that 20:4 level in TAG fraction was noticeably elevated in the C + CBD, HFD and HFD + CBD in relation to the control group (C + CBD: + 145.48%, HFD: + 1047.69%, HFD + CBD: + 968.41%, *p* < *0.0001*, Fig. 1C).

### The activity of the n-3 pathway in selected lipid fractions

**Fig. 2:**
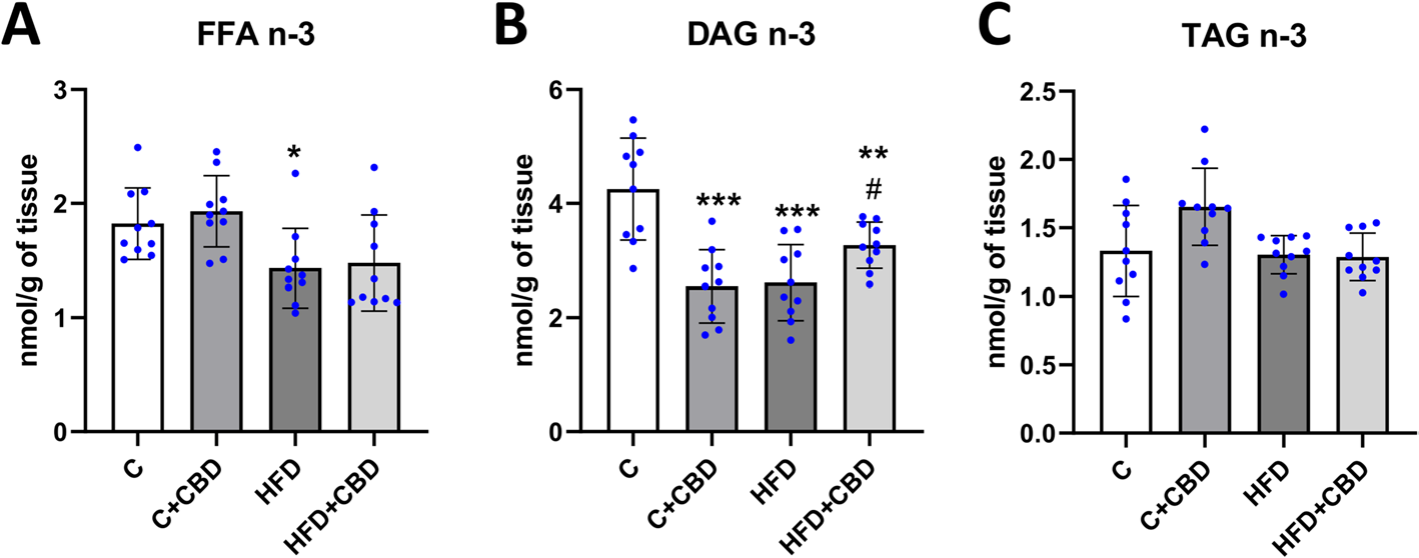
The activity of the omega-3 fatty acid pathway. The activity of the n-3 pathway calculated from the concentration of eicosapentaenoic acid (20:5), docosahexaenoic acid (22:6), and alfa linolenic acid (18:3 n-3) in (**A**) FFA—free fatty acid; (**B**) DAG – diacylglycerol; (**C**) TAG – triacylglycerol fractions in the liver tissue after intraperitoneal injections of CBD during the last 2 weeks of the experiment to rats fed a standard diet (C) or high-fat diet (HFD) for 7 weeks. The concentrations of selected fatty acids used for calculations were measured by the GLC method. The data is presented as mean values ± standard deviation (SD) based on ten independent determinations in each experimental group. *–p < 0.05; **–p < 0.01; ***–p < 0.001; ****–p < *0.0001* significant change vs control group; *#–p* < *0.05; ##–p* < *0.01; ###–p* < *0.001; ####–p* < *0.0001* significant change vs HFD group

In the present study it was observed that rats fed with a high-fat diet had markedly lowered activity of the n-3 pathway in FFA fraction relative to the control group (HFD: −21.40%, *p* < *0.05*, Fig. 2A). Moreover, in DAG fraction a significant decrease was detected in all the groups in comparison with the control group (C + CBD: −40.08%, HFD: −38.56%, *p* < *0.001*, HFD + CBD: −23.13%, *p* < *0.01*, Fig. 2B) while compared to the HFD group, the activity of the n-3 pathway in HFD + CBD group was substantially increased (HFD + CBD: + 25.11%, *p* < *0.05*, Fig. 2B). In contrast, there was a lack of any remarkable changes in the activity of the selected n-3 pathway in TAG fraction (*p* < *0.05*, Fig. 2C).

### The activity of the n-6 pathway in selected lipid fractions

**Fig. 3:**
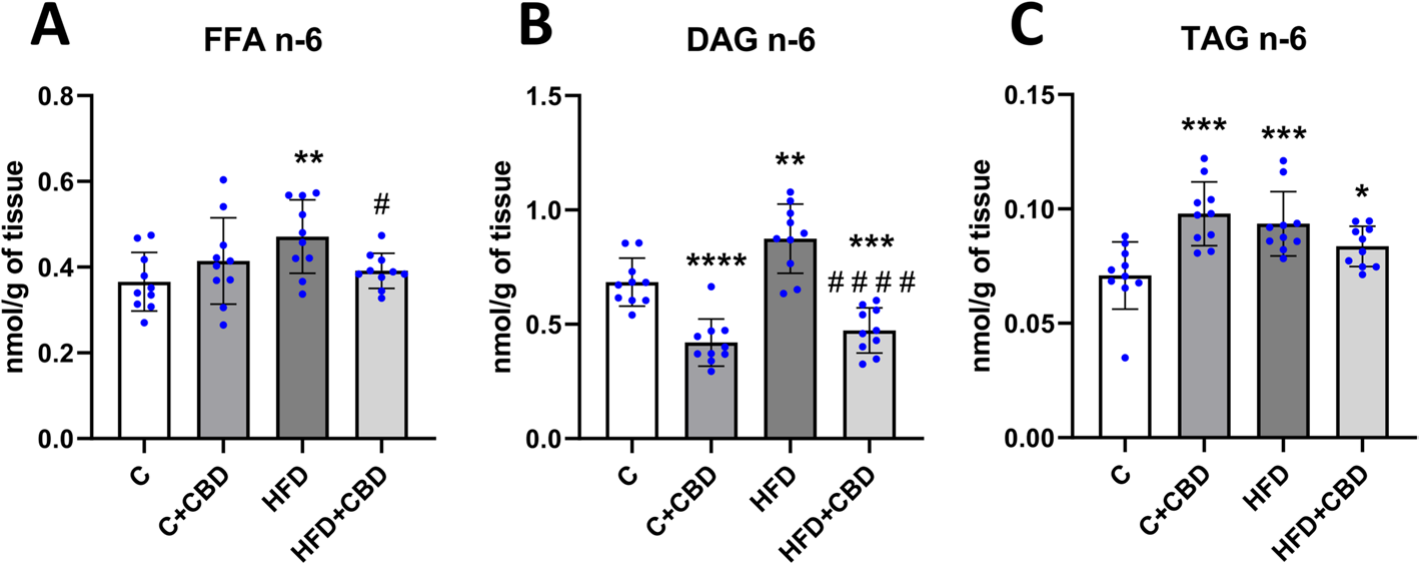
The activity of the omega-6 fatty acid pathway. The activity of the *n*−6 pathway calculated from the concentration of arachidonic acid (AA, 20:4) and linoleic acid (18:2) in (**A**) FFA—free fatty acid; (**B**) DAG – diacylglycerol; (**C**) TAG – triacylglycerol fractions in the liver tissue after intraperitoneal injections of CBD during the last 2 weeks of the experiment to rats fed a standard diet (C) or high-fat diet (HFD) for 7 weeks. The concentrations of selected fatty acids used for calculations were measured by the GLC method. The data is presented as mean values ± standard deviation (SD) based on ten independent determinations in each experimental group. *–p < 0.05; **–p < 0.01; ***–p < 0.001; ****–p < *0.0001* significant change vs control group; *#–p* < *0.05; ##–p* < *0.01; ###–p* < *0.001; ####–p* < *0.0001* significant change vs HFD group

Rats fed with the HFD had significantly increased activity of the n-6 pathway in the FFA fraction in comparison with the group nourished solely with a control diet (HFD: + 28.82%, *p* < *0.01*, Fig. 3A). Furthermore, there was a significant decrease in HFD + CBD group compared to the HFD group (HFD + CBD: −16.95%, *p* < *0.05*, Fig. 3A). Animals fed a HFD in comparison with the control group had eminently increased activity of the n-6 pathway in the DAG fraction (HFD: + 27.71%, *p* < *0.01*, Fig. 3B). In contrast, rats from C + CBD group as well as those from HFD + CBD group had markedly decreased activity of this pathway in the DAG fraction compared to the control group (C + CBD: −38.63%, *p* < *0.0001*, HFD + CBD: −30.88%, *p* < *0.001*, Fig. 3B). Moreover, in HFD + CBD group significantly lowered activity of the n-6 pathway in DAG fraction in relation to the HFD group was noticed. (HFD + CBD: −45.88%, *p* < *0.0001*, Fig. 3B). In the TAG fraction, however, a significant increase was spotted in the activity of the n-6 pathway in all the examined groups in comparison with the control group. (C + CBD: + 42.25%, HFD: + 35.93%, *p* < *0.001*, HFD + CBD: + 22.99%, *p* < *0.05*, Fig. 3C).

### The expression of the enzymes involved in the synthesis of eicosanoids

**Fig. 4:**
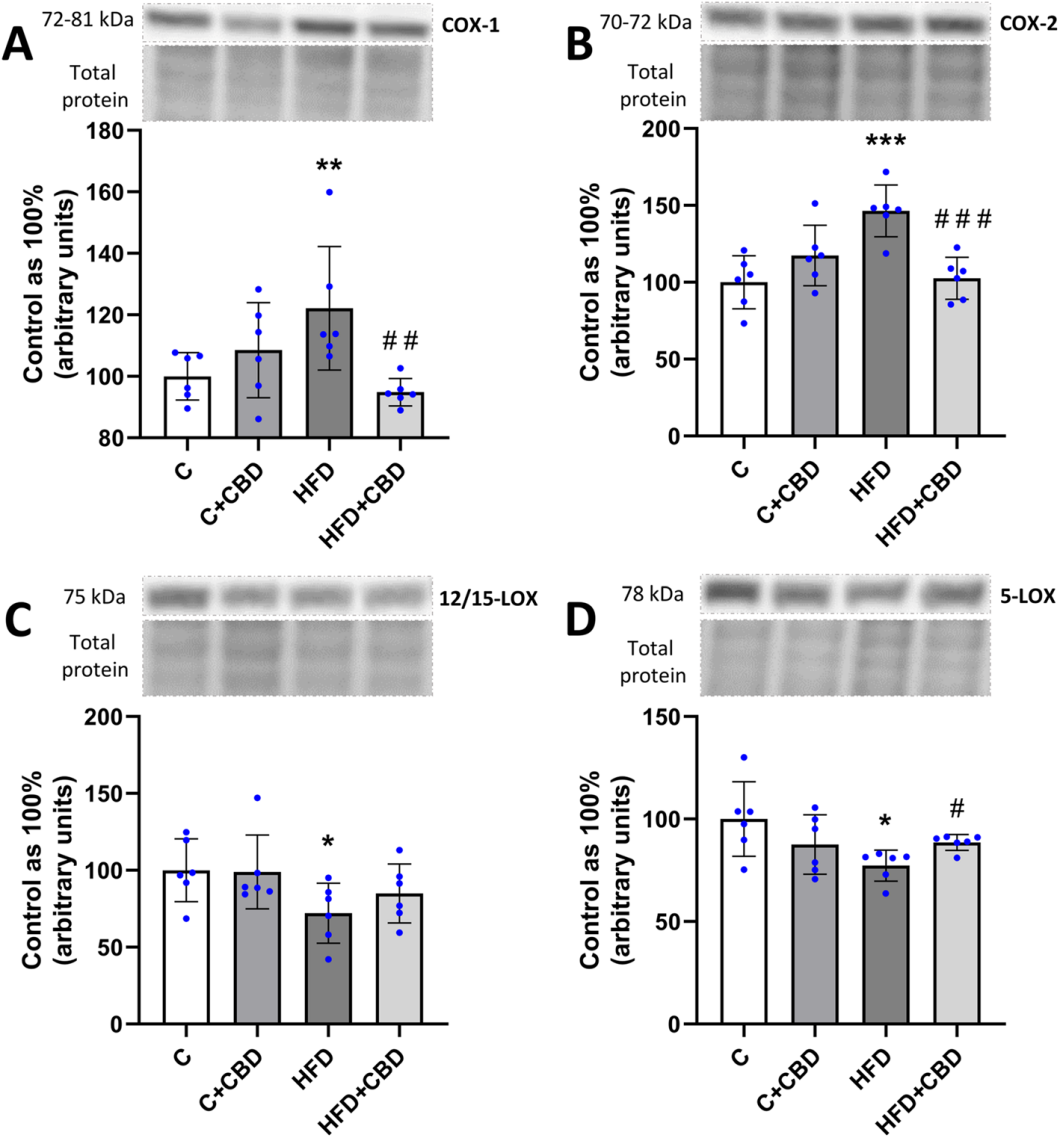
The expression of enzymes from the eicosanoid and prostanoid pathways. The expression of (**A**) COX-1—cyclooxygenase 1; (**B**) COX-2 – cyclooxygenase 2; (**C**) 5-LOX – 5-lipoxygenase; (**D**) 12/15-LOX –12/15-lipoxygenase in the liver tissue after intraperitoneal injections of CBD during the last 2 weeks of the experiment to rats fed a standard diet (C) or high-fat diet (HFD) for 7 weeks. The Western blotting technique was used to measure the enzymes’ expression. The overall expression of the enzymes is displayed as a percentage difference, with 100% being the control group. The data is presented as mean values ± standard deviation (SD) based on six independent determinations in each experimental group. *–p < 0.05; **–p < 0.01; ***–p < 0.001; ****–p < *0.0001* significant change vs control group; *#–p* < *0.05; ##–p* < *0.01; ###–p* < *0.001; ####–p* < *0.0001* significant change vs HFD group

The expression of COX-1 assessed by Western Blotting was significantly increased in the HFD group compared to the control group (HFD: + 22.14%, *p* < *0.01*, Fig. 4A) and decreased in HFD + CBD group in comparison with the HFD group (HFD + CBD: −22.43%, *p* < *0.01*, Fig. 4A). Likewise, similar changes were observed in the expression of COX-2 in the HFD as well as HFD + CBD groups (HFD: + 46.42%, *p* < *0.001*, HFD + CBD: −29.94%, *p* < *0.001*, Fig. 4B). Moreover, it was noticed that the expression of 12/15-LOX was considerably lowered in the HFD group in relation to the control group (HFD: −32.94%, *p* < *0.05*, Fig. 4C), as well as the expression of 5-LOX (HFD: −22.74%, *p* < *0.05*, Fig. 4D). Additionally, a substantial increase in the 5-LOX expression was spotted in the HFD + CBD group in comparison with the HFD group (HFD + CBD: + 14.47%, *p* < *0.05*, Fig. 4D).

### The liver histology and function, as well as marker of oxidative stress development

**Fig. 5:**
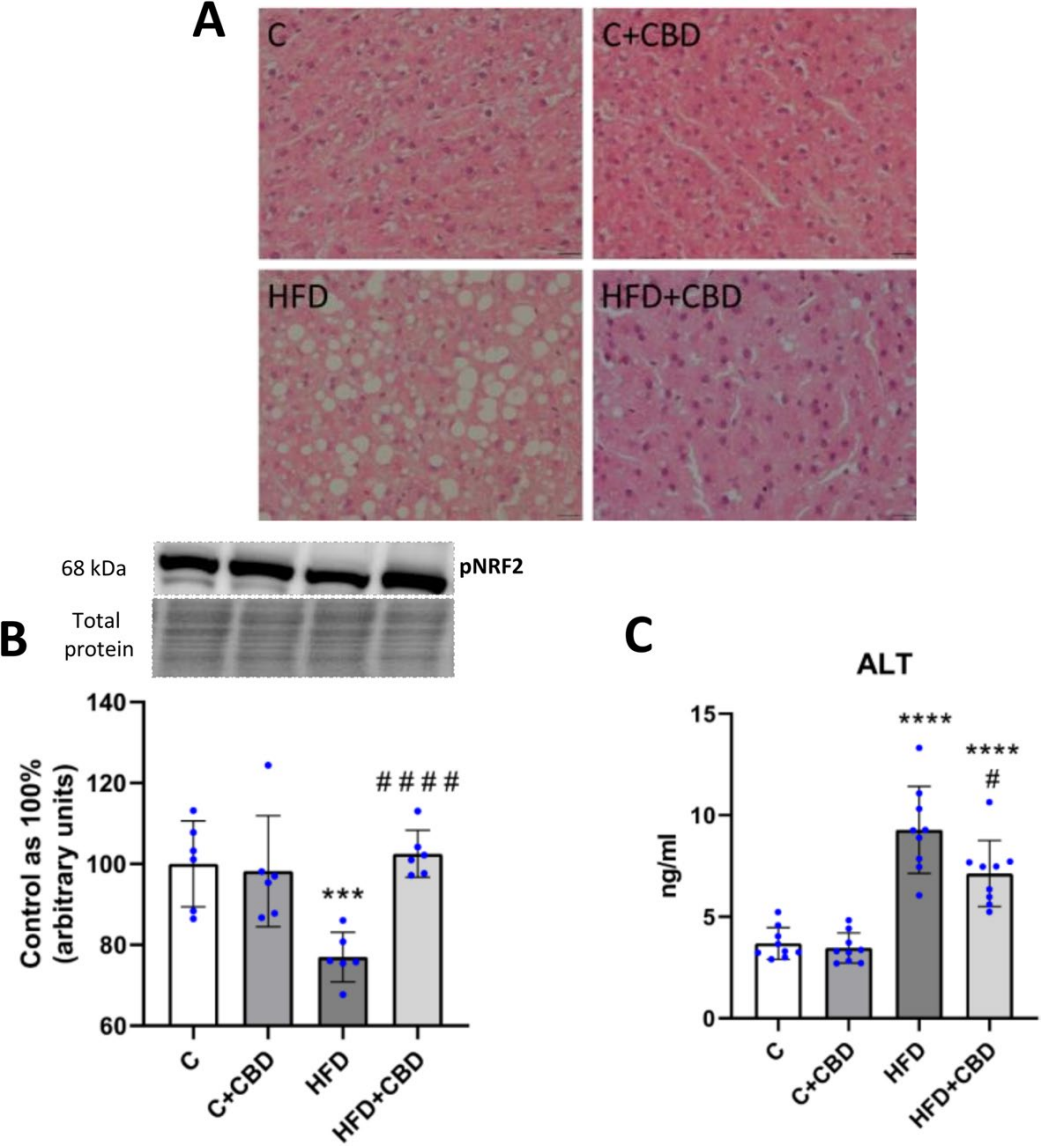
The assessment of liver function and oxidative stress marker. The representative (**A**) histological images of the liver, (**B**) the liver expression of pNRF2(Ser40) – phosphorylated at Serine 40 Nuclear factor erythroid 2-related factor 2; and (**C**) plasmatic concentration of ALT – alanine aminotransferase after intraperitoneal injections of CBD during the last 2 weeks of the experiment to rats fed a standard diet (C) or high-fat diet (HFD) for 7 weeks. Western blotting technique was used to measure the proteins’ expression, and the enzymes’ concentration was measured with an ELISA kit. The overall expression of the protein is displayed as a percentage difference, with 100% being the control group, and ALT concentration is expressed in ng per ml of plasma. In the histological images showing hematoxylin–eosin staining of the hepatic tissue, scale bars are 20 µm. The data is presented as mean values ± standard deviation (SD) based on six (pNRF2, histology) or nine (ALT) independent determinations in each experimental group. *–p < 0.05; **–p < 0.01; ***–p < 0.001; ****–p < *0.0001 significant change vs control group; #–p* < *0.05; ##–p* < *0.01; ###–p* < *0.001; ####–p* < *0.0001* significant change vs HFD group

Histopathological evaluation of the liver from rats fed a high-fat diet indicated increased macrovesicular steatosis compared to the liver of control animals. After simultaneous treatment with a high-fat diet and CBD, the condition of hepatocytes was improved, which was observed on hematoxylin and eosin staining images as a decreased amount of large, clear vacuoles, which are left after lipid deposits (Fig. 5A).

The expression of pNRF2 (Ser40) was significantly decreased after HFD feeding compared to the control group (HFD: −23.05%, *p* < *0.001*, Fig. 5B). However, CBD treatment increased the expression of pNRF2 compared to the HFD-fed animals (HFD + CBD: + 33.22%, *p* < *0.0001*, Fig. 5B). Moreover, to assess precisely the liver function, the ALT concentration in the plasma was measured. It was observed that the enzyme content was significantly increased in the plasma of animals fed an HFD compared to animals fed a standard diet (HFD: + 151.42%, *p* < *0.0001*, Fig. 5C). The HFD group treated with CBD showed a notably increased concentration of ALT compared to the control animals (HFD + CBD + 93.21%, *p* < *0.0001*, Fig. 5C), and it was decreased when compared with the HFD group (HFD + CBD −23.15%, *p* < *0.05*, Fig. 5C).

### The concentrations of the chemokines and cytokines

In Table 1, the concentrations of the chosen cytokines and chemokines measured in the liver tissue are shown. The only detected change was seen in the concentration of RANTES (−36.06%, *p* < *0.05*), which was significantly decreased in the C + CBD group in relation to the control group. However, an increase was found in the concentrations of IL-1β (+ 22.99%, *p* < *0.01*), IFN-γ (+ 23.40%, *p* < *0.001*), TNF-α (+ 21.24%, *p* < *0.01*), IL-2 (+ 29.80%, *p* < *0.05*), IL-7 (+ 29.96%, *p* < *0.001*), IL-12p70 (+ 28.31%, *p* < *0.01*), IL-18 (+ 11.84%, *p* < *0.01*), GM-CSF (+ 30.95%, *p* < *0.01*), IL-10 (+ 19.25%, *p* < *0.05*) in rats fed a HFD compared to the control group. Similarly, in animals exposed simultaneously to HFD and CBD a remarkable elevations were found in the concentrations of: IL-1α (+ 35.05%, *p* < *0.01*), IL-5 (+ 37.29%, *p* < *0.001*), IL-6 (+ 21.11%, *p* < *0.01*), IFN-γ (+ 18.84%, *p* < *0.05*), IL-12p70 (+ 21.82%, *p* < *0.05*), IL-17 (+ 87.29%, *p* < *0.01*), G-CSF (+ 50.75%, *p* < *0.01*), M-CSF (+ 56.42%, *p* < *0.05*), VEGF (+ 72.60%, *p* < *0.01*), IL-4 (+ 39.10%, *p* < *0.05*), IL-13 (+ 35.47%, *p* < *0.05*), in comparison with the control group. Moreover, the content of IL-1α (+ 23.91%, *p* < *0.01*), IL-5 (47.30%, *p* < *0.001*), IL-17 (+ 87.29%, *p* < *0.05*), G-CSF (35.08%, *p* < *0.0001*), M-CSF (+ 47.62%, *p* < *0.05*), VEGF (+ 44.52%, *p* < *0.05*), IL-4 (+ 46.62%, *p* < *0.01*), IL-13 (+ 71.85, *p* < *0.05*), was significantly enhanced while the concentration of IL-2 (−40.17%, *p* < *0.01*), IL-7 (−20.63%, *p* < *0.05*), GM-CSF (−22.73%, *p* < *0.05*), IL-10 (−16.16%, *p* < *0.05*) and RANTES (−46.02%, *p* < *0.05*) was markedly decreased in HFD + CBD group in comparison with the HFD group.

**Table 1:** The concentration of chosen chemokines and cytokines

Protein (pg/ml)		**C**	**C + CBD**	**HFD**	**HFD + CBD**
Pro-inflammatory	**IL-1α**	277.01 ± 38.34	294.66 ± 62.52	301.93 ± 31.50	374.11 ± 34.54 ** ##
	**IL-1β**	182.55 ± 20.33	187.40 ± 23.04	224.51 ± 20.33 **	192.96 ± 30.00
	**IL-5**	6852.02 ± 865.08	6089.91 ± 655.40	6386.47 ± 679.77	9407.14 ± 232.46 *** ####
	**IL-6**	1724.49 ± 179.68	1740.26 ± 106.69	1800.50 ± 264.75	2088.59 ± 204.79 **
	**IFN-γ**	922.86 ± 84.51	908.38 ± 86.15	1138.79 ± 84.51 ***	1096.74 ± 123.42 *
	**TNF-α**	1980.29 ± 144.09	1866.02 ± 127.19	2400.88 ± 270.91 **	2154.23 ± 210.66
Bacterial/viral	**IL-2**	14,920.11 ± 3248.20	15,256.07 ± 3714.66	19,366.18 ± 2915.61 *	11,587.75 ± 2954.48 ##
	**IL-7**	532.94 ± 32.99	511.99 ± 70.46	692.60 ± 80.02 ***	549.72 ± 88.28 #
	**IL-12p70**	1158.71 ± 141.09	1162.33 ± 178.96	1486.77 ± 191.55 **	1411.56 ± 182.95 *
	**IL-17**	176.18 ± 68.74	170.80 ± 50.54	180.58 ± 80.57	329.95 ± 86.63 ** #
	**IL-18**	8947.55 ± 370.84	8690.74 ± 510.47	10,006.98 ± 638.16 **	9455.22 ± 485.09
Macrophage-associated	**G-CSF**	7.39 ± 1.61	6.67 ± 0.75	8.25 ± 1.55	11.14 ± 1.40 ** ####
	**GM-CSF**	368.49 ± 19.94	363.34 ± 52.36	482.53 ± 63.20 **	372.83 ± 58.68 #
	**M-CSF**	23.31 ± 7.15	20.41 ± 5.65	24.70 ± 7.94	36.46 ± 8.51 * #
	**MCP-1**	745.31 ± 55.16	727.63 ± 30.05	794.04 ± 111.73	824.69 ± 75.18
	**MIP-1α**	60.87 ± 15.56	82.18 ± 19.30	72.39 ± 19.73	82.01 ± 17.65
	**MIP-3α**	65.97 ± 20.55	93.01 ± 25.01	60.08 ± 12.87	69.17 ± 26.92
	**RANTES**	1101.94 ± 273.78	704.61 ± 158.78 *	1313.86 ± 153.91	709.23 ± 332.89 #
	**VEGF**	82.30 ± 23.48	84.98 ± 14.10	98.29 ± 26.08	142.05 ± 30.40 ** #
Anti-inflammatory	**IL-4**	8060.94 ± 2023.64	7940.41 ± 1627.26	7647.87 ± 2053.18	11,212.97 ± 1326.80 * ##
	**IL-10**	681.69 ± 54.40	654.58 ± 76.62	812.93 ± 96.61 *	681.53 ± 55.63 #
	**IL-13**	1441.40 ± 344.39	1255.19 ± 361.36	1136.29 ± 592.23	1952.73 ± 148.15 * #

The concentration of chosen chemokines and cytokines: granulocyte colony-stimulating factor (G-CSF), granulocyte–macrophage colony-stimulating factor (GM-CSF), growth-regulated oncogenes/keratinocyte chemoattractant (GRO/KC), interleukin 1α (IL-1α), interleukin 1β (IL-1β), interleukin 2 (IL-2), interleukin 4 (IL-4), interleukin 5 (IL-5), interleukin 6 (IL-6), interleukin 7 (IL-7), interleukin 10 (IL-10), interleukin 12p70 (IL-12p70), interleukin 13 (IL-13), interleukin 17 A (IL-17 A), interleukin 18 (IL-18), interferon γ (IFN-γ), macrophage colony-stimulating factor (M-CSF), macrophage inflammatory protein 1α (MIP-1α), macrophage inflammatory protein 3α (MIP-3α), monocyte chemoattractant protein-1 (MCP-1), regulated on activation, normal T-cell expressed and secreted (RANTES), tumor necrosis factor α (TNF-α), vascular endothelial growth factor (VEGF) in the liver tissue after intraperitoneal injections of CBD during the last 2 weeks of experiment to rats fed a standard diet (C) or HFD for 7 weeks. The concentration was measured by Multiplex assay and is expressed in picograms per milliliter of protein. The data is presented as mean values ± standard deviation (SD) based on eight independent determinations in each experimental group. *–p < 0.05; **–p < 0.01; ***–p < 0.001; ****–p < *0.0001* significant change vs control group; *#–p* < *0.05; ##–p* < *0.01; ###–p* < *0.001; ####–p* < *0.0001* significant change vs HFD group

## Discussion

The present study is a continuation of previous work describing CBD as a potential element in the treatment of neurodegenerative diseases. It was observed that CBD exerted a potent ability to diminish the deposition of proinflammatory lipid precursors in the cerebral cortex of rats fed a high-fat diet (Opęchowska et al. 2024). In order to better understand the interplay between CBD treatment and diseases developed under HFD conditions and diet-induced inflammation, the analysis was conducted on the liver, the organ that is said to be the main hub in lipid metabolism. Thus, in the present study, the effect of CBD on early-stage inflammation in MASLD progression caused by an HFD was evaluated. To obtain reliable results, the most effective dose of CBD (10 mg/kg/day) was used (Navarrete et al. 2018; Xu et al. 2019a). Although human studies indicated that purified CBD is well-tolerated at relatively high amounts (6000 mg), some animal studies showed that higher doses of CBD may cause hepatotoxicity, especially in the fed state (Taylor et al. 2018; Lakhani et al. 2023). Moreover, it was indicated in animal models where mice suffered from diabetes that treatment with higher doses of cannabidiol (20 mg/kg of body weight/day) exerted the same or comparable effects as 10 mg of CBD in diabetic heart muscle, where attenuation of nitrative stress, cell death, and fibrosis was observed (Rajesh et al. 2010). In this study, a 2-week treatment was selected to observe very early changes in MASLD exerted by CBD in the liver, just before other systemic effects or liver adaptive mechanisms would be initiated. Moreover, studies where a single 30-mg dose of CBD was used showed that the highest C_max_ of CBD was projected in the liver with 13 times higher concentrations than in plasma (Liu and Sprando 2023). Thus, this may suggest that changes observed in the liver emerged first and proceeded with other systemic effects; however, more studies with longer CBD exposition are needed.

The report indicated that the increased availability of fatty acids present in the diet has led to changes in the fatty acid composition of deposited lipids in liver tissue. In the present study, a decrease in the activity of the n-3 PUFAs pathway was observed in the FFA and DAG fractions in the HFD group, with no significant changes in the TAG pool. According to a study by Echeverría et al. on male mice livers, the HFD diet resulted in a decrease in the content of the two dominant fatty acids in the n-3 PUFA pathway: docosahexaenoic acid (DHA; C22:6n-3) and eicosapentaenoic acid (EPA; C20:5n-3). These findings may explain the decrease in n-3 PUFA activity observed here (Echeverría et al. 2019). Furthermore, in a group of animals fed a high-fat diet with CBD administration, an increase in the activity of this pathway was noted only in the diacylglycerol fraction relative to the HFD-fed group of animals. Although similar changes were not observed in the other pools, the increase in this particular fraction is very significant. It is the most biologically active fraction, likely to interfere with other signaling pathways such as insulin signaling (Zabielski et al. 2018; Błachnio-Zabielska et al. 2012). Additionally, the HFD caused an increment in the activity of the pro-inflammatory n-6 PUFAs pathway in all the examined lipid fractions. These observations are consistent with the findings of another study by Turner et al., which showed that female Sprague–Dawley rats fed an HFD for 8 weeks had markedly higher fatty acid (FA) n-6/n-3 ratios for both white and red skeletal muscles (Turner et al. 2004). In another study by Da Silva-Santi et al., a high-fat diet lasting for 8 weeks increased fat accumulation in the liver of male Swiss mice, and changes in fatty acid composition were observed at time points after 7, 14, 28, and 56 days. In this investigation, the n-6/n-3 fatty acid ratio decreased even though lipid accumulation increased throughout the experiment. The differences between these results and the present study are likely due to differences in diet composition. In the study by Da Silva-Santi et al., the HFD consisted of 34.48% saturated fatty acids (SFA), 41.40% monounsaturated fatty acids (MUFA), and 24.12% PUFA (Silva-Santi et al. 2016). In contrast, the HFD in the present experiment consisted of 72.00% SFA, 16.80% MUFA, and 11.20% PUFA. There are indications that the higher proportion of PUFAs in the HFD used in the cited study, compared to our diet, may have contributed to lower activity of the enzyme delta-6-desaturase (D6D). This enzyme is responsible for converting n-6 fatty acids into more active forms. The reduced activity of D6D may also have contributed to the decrease in the n-6/n-3 ratio observed in the Da Silva-Santi et al. study (Silva-Santi et al. 2016). Although the activity of D6D was not measured in this study, the lower levels of PUFA in the diet used here resulted in increased activity in the n-6 PUFA pathway, which plays an important role in the development of inflammation. The increase in the n-6 PUFA pathway activity is due to an increase in AA levels, as arachidonic acid is the dominant fatty acid of the n-6 pathway (Schmitz and Ecker 2008). In the study presented, an increase in the n-6 PUFA pathway activity in the high-fat diet group correlated with a rise in the AA content in the HFD group in all assessed lipid pools. These findings align with existing literature indicating that an HFD leads to increased production of lipid mediators of inflammation such as AA, thereby enhancing cell signaling associated with the inflammatory process (Sonnweber et al. 2018). Studies conducted by Da Silva-Santi et al., showed that increased lipid accumulation in HFD mice livers correlated with an increase in arachidonic acid content. In this experiment, a reduction in AA was only seen in the last FA composition measurement performed after day 56, which may indicate how the liver tissue has adapted to lipid overload circumstances (Silva-Santi et al. 2016; Xu et al. 2019b). Evaluating the impact of CBD supplementation on the previously mentioned parameters in the HFD-fed animal group, in the present study, a reduction was observed in the activity of the n-6 PUFA pathway as well as AA levels in the FFA and DAG fractions as compared to the HFD group. The effect of CBD on arachidonic acid levels in liver tissue has not been described so far. This may be a consequence of CBD’s inhibitory effect on ROS cellular production, which is consistent with the inhibition of PUFA peroxidation through interaction with membrane lipids (Degrave et al. 2023; Pagano et al. 2023). To confirm these suspicions, the expression of the phosphorylated form of NRF2 at Ser40 was determined. Since this protein is a major regulator of redox balance by promoting the transcription of antioxidant genes, the decrease in NRF2(Ser40) expression observed in the HFD group was restored by CBD (Arroyave-Ospina et al. 2021). Although this at least partially may indicate that CBD exerts an inhibitory effect on ROS production, more studies determining CBD’s effect on oxidative stress markers should be performed with the use of NRF2 knockout animal model fed a high-fat diet. The lipid data mentioned above suggest that CBD alters the accumulated lipid profile associated with the inflammatory process. In all the parameters described above: n-3 and n-6 PUFA pathway activity, AA content in the HFD + CBD group, statistically significant changes were not observed in the TAG pool. In addition, no significant changes in the activity of the n-3 PUFA pathway were observed in the FFA fractions compared to the HFD group. This is most likely due to the transport of fatty acids that are contained in these fractions to other tissues more adapted to fat accumulation than the liver, as reported in the literature. In a study conducted by Teusink et al. on male Wistar rats, appropriate radioactive tracers were used to observe the appearance of oleic acid in plasma. In this way, the researchers presented that fatty acids synthesized in the TAG fraction could be incorporated into lipoproteins to transport them from the site of synthesis to the tissues, where they are oxidized or stored (Griffin 2013; Teusink et al. 2003). This may explain the lack of significant changes in the above parameters in the TAG fraction in the HFD + CBD group. At the same time, there is no significant change in the TAG fraction in n-6 PUFA pathway activity and AA content, and only an apparent decreasing trend shows that TAG is a relatively safe lipid pool for hepatocytes. The lack of significant changes in the activity of the n-3 PUFA pathway in the FFA fraction is probably because fatty acids stored in this fraction in the liver can be transported to other tissues that are more adapted to fat storage, such as adipose tissue. In a study conducted by Baker et al. on male Sprague–Dawley rats, the fate of FFAs in the body was investigated by injecting palmitate-1-^14^C into the tail vein of unanesthetized fasting rats. The authors of the cited study repeatedly mention the ‘recycling’ of FFAs, indicating their continuous exchange between different areas in the body, suggesting that FFAs are an easily exchangeable fraction (Baker and Schotz 1967). The previously mentioned dominant fatty acid in the n-6 PUFA pathway, namely arachidonic acid, when released from the plasma membrane, can be used as a substrate for the synthesis of eicosanoids by the enzymes LOX and COX (CORRECTIONS. 2002). The first pathway of AA metabolism is the cyclooxygenase cycle (Smith and Murphy 2016). There are two isoforms of COX. Cyclooxygenase-1 is constitutively expressed in many types of cells and plays a crucial role in homeostasis. Under baseline conditions, COX2 is often absent. However, it can be induced in various inflammatory conditions and cell damage by various cytokines, growth factors, and mitogens (Hu 2003; Fosslien 2000). The expression of COX-1 is linked to the creation of thromboxane (TX) and cytosolic prostaglandin E2 (PGE2), whereas COX-2 plays a crucial role in producing microsomal prostaglandin E2 and prostaglandin I2 (PGI2) (Harizi et al. 2008; Hanna and Hafez 2018). The changes in arachidonic acid levels in the HFD and HFD + CBD groups that were discussed above were reflected in changes in COX-1 and COX-2 expression. In the present study, the increase in AA levels in the FFA fraction and DAG correlates with the increase in COX-1 and COX-2 expression in the HFD group. The above change indicates that in this experiment, an inflammatory process was developed at an early stage in MASLD progression, as not all assessed pro-inflammatory cytokines showed an increase after HFD. Importantly, CBD supplementation to rats fed a high-fat diet decreased both COX-1 and COX-2 expressions to a similar level as AA content in the FFA and DAG fractions. Consistent effects of CBD on COX expression in liver tissue have been noted by Ma et al. on a mouse model of liver fibrosis induced with carbon tetrachloride (CCL4) for 10 weeks. They reported that CBD treatment resulted in a significant decrease in COX-2 expression (Ma et al. 2024). Both studies, cited above and present, indicated that CBD affects early indicators of inflammation by effectively mitigating lipid precursors of inflammation in the liver. The second pathway of AA metabolism is the lipoxygenase cycle (Smith and Murphy 2016). Lipoxygenases are enzymes that facilitate the transformation of polyunsaturated fatty acids, particularly arachidonic acid and linoleic acid found in the plasma membrane, into oxidized lipids that contribute to inflammatory processes (Heinrich et al. 2022). The pro-inflammatory effects of 5-LOX in liver tissue have also been documented by other researchers. In a study by Ma et al. on a rat model of the progression of NAFLD induced by consuming HFD, the researchers presented the relationship between an increase in the concentration of substrates and products of the AA/5-LOX pathway and NAFLD progression (Ma et al. 2017). In another study by Peltner et al. in zymosan-induced murine peritonitis, it was observed that CBD caused a lipid mediator class switch: it inhibited 5-LOX and consequently reduced the biosynthesis of pro-inflammatory leukotrienes and activated 12/15-LOX, which resulted in increased production of anti-inflammatory products (Peltner et al. 2023). In the present study, it was observed that 5-LOX and 12/15-LOX expressions were decreased in the liver tissue of rats fed a high-fat diet. After cannabidiol treatment, a significant increment was noticed in the 5-LOX expression, and surprisingly, a lack of significant change in the 12/15-LOX expression. The most possible explanation for these results may be the fact that 5-LOX is responsible for the synthesis of the first AA derivatives, i.e., hydroperoxyacosatetraenoic acid (5-HPETE), which can inhibit 12/15-LOX activity (Kotlyarov 2022). However, herein, the 5-HPETE level was not assessed, which seems to be another weakness of the present study. In contrast, Takeda et al. in their experiment noted that CBD inhibits both 5-LOX and 15-LOX, but 15-LOX activity is more selectively inhibited by CBD than 5-LOX (Takeda et al. 2009). This discrepancy could be because the present study was conducted on animals, while the previously mentioned author conducted his study on an in vitro model using purified 5-LOX and 15-LOX enzymes. The authors did not investigate the effects of these compounds on hepatocytes or other specific cell types.

To correlate the effect of CBD on lipid precursors of inflammation with markers of inflammation development, cytokine and chemokine production were analyzed. In the present study, the aim was to assess the effect of CBD on the progression from HFD-induced MASLD to MASH; only a portion of the cytokine and chemokine levels associated with inflammation development were affected in the HFD group. A notable example is the increase in TNFα content, while the IL-6 level remained unchanged. However, CBD treatment induced significant and sometimes opposite changes in many of the assessed cytokines and chemokines.

In this study, within a group of cytokines associated with the stimulation of the proliferation and differentiation of hematopoietic progenitor cells, CBD treatment in the HFD group resulted in various effects. Firstly, there was a significant decrease in GM-CSF levels observed in the HFD-fed animals with CBD treatment. A similar effect of CBD on this parameter was also reported in a study by Rachayon et al. (Rachayon et al. 2022). According to the literature, a decrease in GM-CSF expression inhibits the progression of liver disease by having an inhibitory effect on fibrosis (Tan-Garcia et al. 2020). This is because a high level of GM-CSF stimulates the development of cells belonging to the granulocytic and macrophage lineage and acts on NK cells to stimulate their cytotoxicity (Hamilton 2020). Secondly, an opposite effect exerted by CBD in the HFD group was a significant increase in the content of G-CSF and M-CSF compared to the HFD group. A similar effect was reported by Hegde et al. in their study, which tested the effect of CBD on the immune system of mice that had not yet been exposed to pathogens. Hegde et al. observed that CBD administration to female mice induced the production of G-CSF and M-CSF, which caused myeloid-derived suppressor cells (MDSC) mobilization resulting in the ability to suppress T cell responses (Hegde et al. 2015). Thus, targeting G-CSF and M-CSF by CBD in the present and cited studies indicates that this cannabinoid exerts immunosuppressive effects induced by selected chemokines.

Another indication of CBD’s immunosuppressive abilities is the decreased concentration of RANTES in the HFD + CBD group compared to the HFD. According to the literature, RANTES plays a role in the progression of liver disease by stimulating T-cell proliferation and activating the cytotoxicity of Tc and NK cells (Mohs et al. 2017). Therefore, a decrease in its level induced by CBD in the HFD group may suggest its immunosuppressive effects and hold promise for future liver disease treatments. The results obtained in this study are consistent with those of Yeisley et al., where CBD-treated activated human macrophages also reduced the expression of RANTES (Yeisley et al. 2021).

Among cytokines that mediate and amplify inflammatory responses, CBD supplementation led to a significant decrease in the level of IL-7 and IL-2 in the HFD group compared to the HFD animals. Alterations in IL-7 content are noteworthy as IL-7 is crucial for the functioning of the immune system, particularly in the proliferation and survival of lymphocytes. Sawa et al., investigating the effect of IL-7 produced by mouse hepatocytes on the T-cell response, noted that increased IL-7 expression correlated with greater activation of the immune response by increasing the number of CD4 + T cells and CD8 + T cells. Furthermore, the researchers observed that decreased IL-7 expression in the liver had the opposite effect, potentially suppressing the immune response and exerting an anti-inflammatory effect (Sawa et al. 2009). The alteration in IL-7 expression following CBD supplementation in this study is consistent with the results of a study by Anil et al. The cited study examined the anti-inflammatory effects of cannabis compounds, including CBD, in COVID-19-associated inflammation in the lung cancer cell line A549 (ATCC® CCL-185™) and macrophage cell line KG1 (ATCC® CCL-246™). The study cited above, along with the present study showed that CBD treatment led to a reduction in IL-7 expression (Anil et al. 2021). Although the role of IL-2 in the inflammatory process is dual and opposite, it has been shown that IL-2 stimulates the rapid proliferation and activation of effector T cells (Th1 and Th2) and Natural Killer cells, amplifying the initial immune response. Moreover, both IL-2 and IL-7 were found to have similar activating effects on T cells in vitro (Katzman et al. 2011).

Therefore, the decrease in the levels of both cytokines induced by CBD in the HFD-fed animals, with a more pronounced effect on IL-2, appears to be significant in demonstrating the specific anti-inflammatory effect of CBD.

What was quite surprising in the obtained results is the fact that CBD did not change the content of TNF-α, IL-12p70, IL-6, and IL-18 in the HFD-fed animals. As TNF-α is the most prominent indicator of inflammation, its content in the HFD group was increased. The lack of significant changes in its content after CBD supplementation is probably due to the relatively small dose of CBD used in the present experiment, although it was based on previous literature. According to a study by Gallily et al. on a zymosan-induced inflammation model in mice, the effect of CBD on TNF-α may be dose-dependent. Researchers analyzing serum TNF-α levels proved that both too low and too high doses of CBD showed no significant effect on TNF-α production. In the present experiment, CBD injections were used at doses of 10 mg/kg of body weight, while researchers from The Hebrew University of Jerusalem found that the maximum inhibitory effect on TNF-α was achieved at 5 mg/kg of body weight (Gallily et al. 2015). However, another study examining the dose-dependent effect of CBD on various pro-inflammatory cytokines and chemokines in the model of diet-induced MASLD is still needed. Additionally, CBD supplementation did not significantly affect IL-18 levels, which is contradictory to studies conducted by Urlic et al., who observed a decrease in IL-18. The discrepancy in the present results is likely due to the fact that the cited study focused on patients with primary hypertension, a condition that was not present in the animal model used in this paper (Urlic et al. 2023). Thus, it may be suggested that the anti-inflammatory effect exerted by CBD depends on the primary cause and condition of the disease. This may also be the explanation for the discrepancy observed in the present study and in Yeisley et al., who noted an inhibitory effect of CBD on VEGF, while in this study, the effect was opposite (Yeisley et al. 2021).

Another significant change exerted by CBD in the HFD group was seen in the group of cytokines belonging to the type 2 immune response. In addition, the increase in IL-4, IL-5, and IL-13 levels after CBD supplementation in the present study is significant because, according to the literature, increases in IL-4, IL-5, and IL-13 are correlated with inhibition of excessive neutrophil recruitment, production of pro-inflammatory cytokines, and liver damage (Seki et al. 2012). Similar results were obtained in the study by Vuolo et al., conducted on adult male Wistar rats in an animal model of asthma, in which CBD treatment reduced cytokine levels such as IL-4, IL-5, and IL-13 (Vuolo et al. 2015). Cannabidiol acting in asthma, a disease associated with an over-reactive immune system, by reducing cytokines, confirming its anti-inflammatory effects. Moreover, IL-4 and IL-13, secreted by Th2-polarized T cells, granulocytes, and monocytes/macrophages, are crucial for tissue repair by activating fibroblasts to promote collagen secretion. Moreover, these cytokines also stimulate macrophages to release growth factors (Grünig et al. 1979; Cordero-Espinoza and Huch 2018). Thus, observed in the present study, enhancement in the level of IL-4 and IL-13 indicates that CBD, through intensified release of these cytokines, may be relevant in tissue repair.

In this study, in the high-fat diet-induced MASLD, CBD treatment is shown to be the most effective in immunosuppression and type 2 immune response. The effects of CBD may depend on the dose and stage of disease progression, making this study preliminary in terms of clinical implications and requiring further examination. There are still uncertainties regarding the precise mechanism of CBD action in liver inflammation. Thus, many studies cited in this paper are based on experiments conducted on different tissues such as the brain or skeletal muscles. Since the disease development mechanisms of each tissue are different, it can only be speculated that the changes observed with CBD would be similar. However, the lack of comparison with other studies describing CBD’s effects in various hepatic models may be an important limitation of this study.

## Conclusion

Consuming a diet high in fats leads to an increased presence of fatty acids, which are used to create substances that cause inflammation. Therefore, such a diet is closely associated with the development of inflammatory conditions. This study demonstrates that cannabidiol effectively mitigates lipid precursors of inflammation in the liver. Cannabidiol’s action is evidenced by a decrease in the activity of the pro-inflammatory n-6 PUFAs pathway in FFA and DAG fractions, while simultaneously increasing the activity of the anti-inflammatory n-3 PUFAs pathway in the DAG pool. The decrease in the level of n-6 PUFA is associated with decreased arachidonic acid deposition in FFA and DAG after CBD supplementation. This seems to be the most important outcome of the present study, as AA is a key precursor of inflammatory mediators. Moreover, CBD significantly affects DAG fractions, which are signaling molecules interacting with many signaling pathways. This fact is extremely important for the pathogenesis of various diseases, such as insulin resistance. Additionally, CBD influences the expression of enzymes involved in arachidonic acid metabolism, namely COX-1 and COX-2, with a concomitant increase in the levels of selected anti-inflammatory cytokines and a decrease in pro-inflammatory cytokines. These findings suggest that CBD exerts a protective effect against the progression of liver inflammation and damage in the very early stages of MASLD deterioration in an animal model. Although the presented study adds some important information about CBD’s potential to become a promising therapeutic avenue for conditions related to MASLD and inflammation, it is of a preclinical nature. Thus, the implications of CBD use in patients suffering from MASLD should be analyzed in future human studies.

## Supplementary Information

Supplementary Material 1

## Data Availability

The data presented in this study are available on request from the corresponding author.
