# Supplementary material for: Therapeutic potential of cannabidiol supplementation in mitigating lipid precursors of inflammation in hepatic steatosis progression
Source: J Cannabis Res. 2026 Feb 25;8:47. doi: 10.1186/s42238-026-00413-z (PMC13041381; doi:10.1186/s42238-026-00413-z)
Supplement: Supplementary file 1 — Supplementary Material 1 [file 42238_2026_413_MOESM1_ESM.pdf]

Original images for blots

The upper images represent the uncropped chemiluminescent blot image corresponding with the images placed in the manuscript.

The lower images represent the stain-free blot image of the same membrane after transfer which is used for creating a multichannel image (overlaid view) to normalize data.

Loading order: control (C), control+cannabidiol (C+CBD), high-fat diet (HFD), high-fat diet + cannabidiol (HFD+CBD).

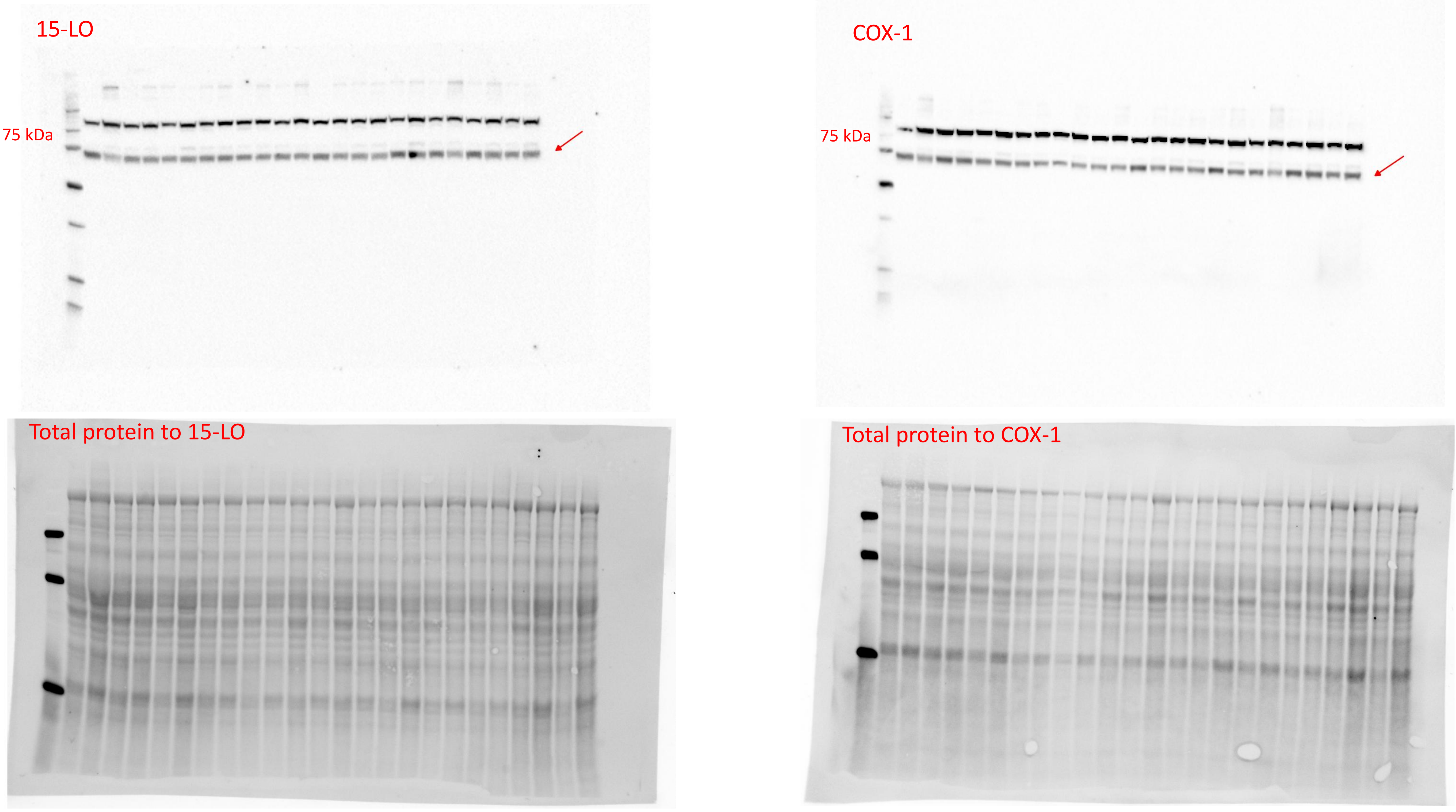
15-LO
75 kDa
COX-1
75 kDa
Total protein to 15-LO
Total protein to COX-1

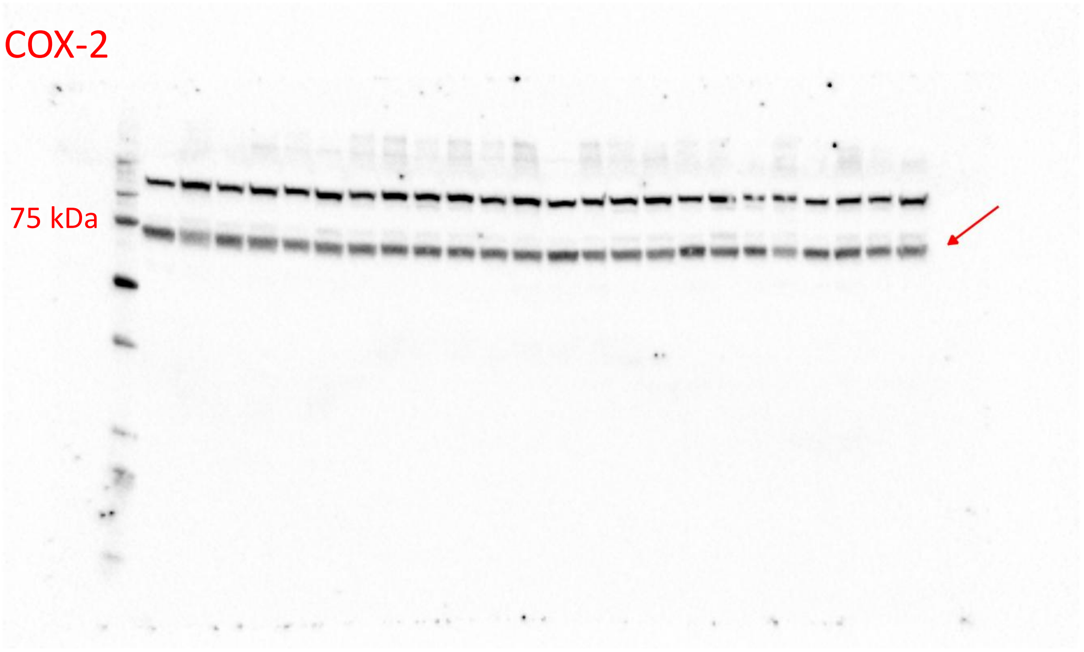
COX-2
75 kDa

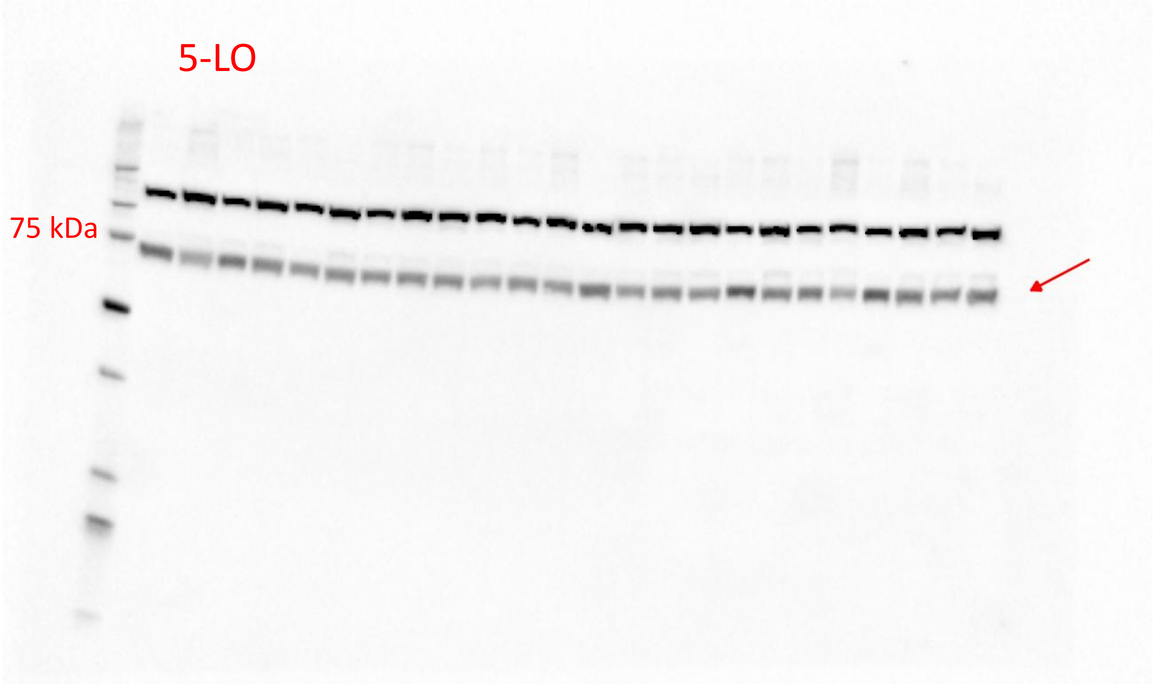
5-LO
75 kDa

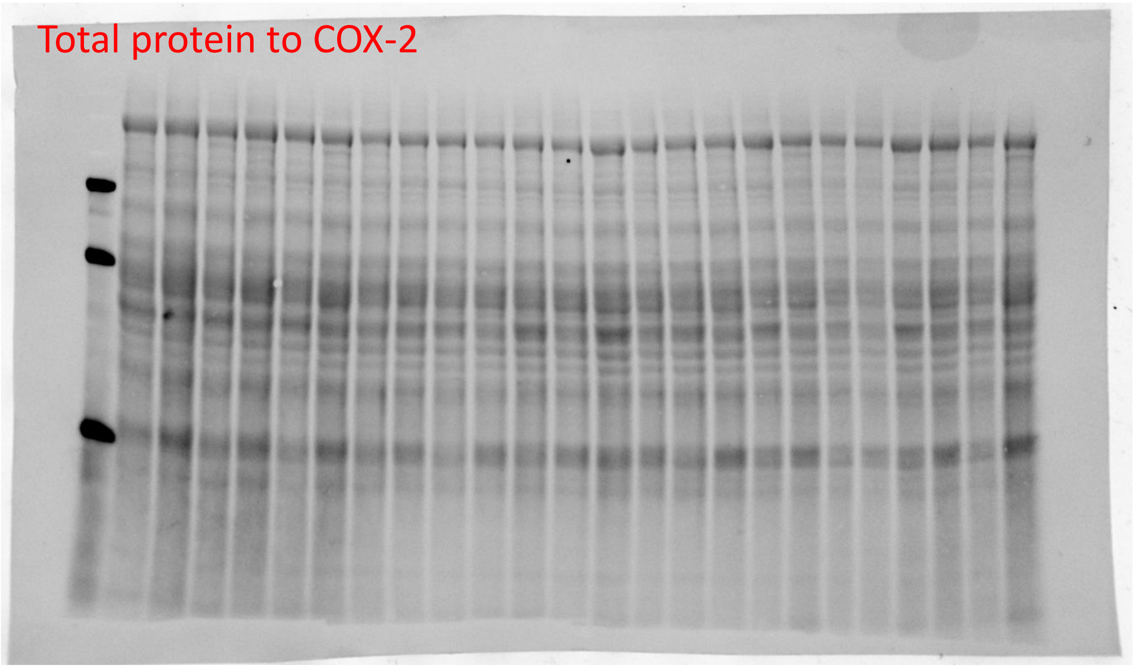
Total protein to COX-2

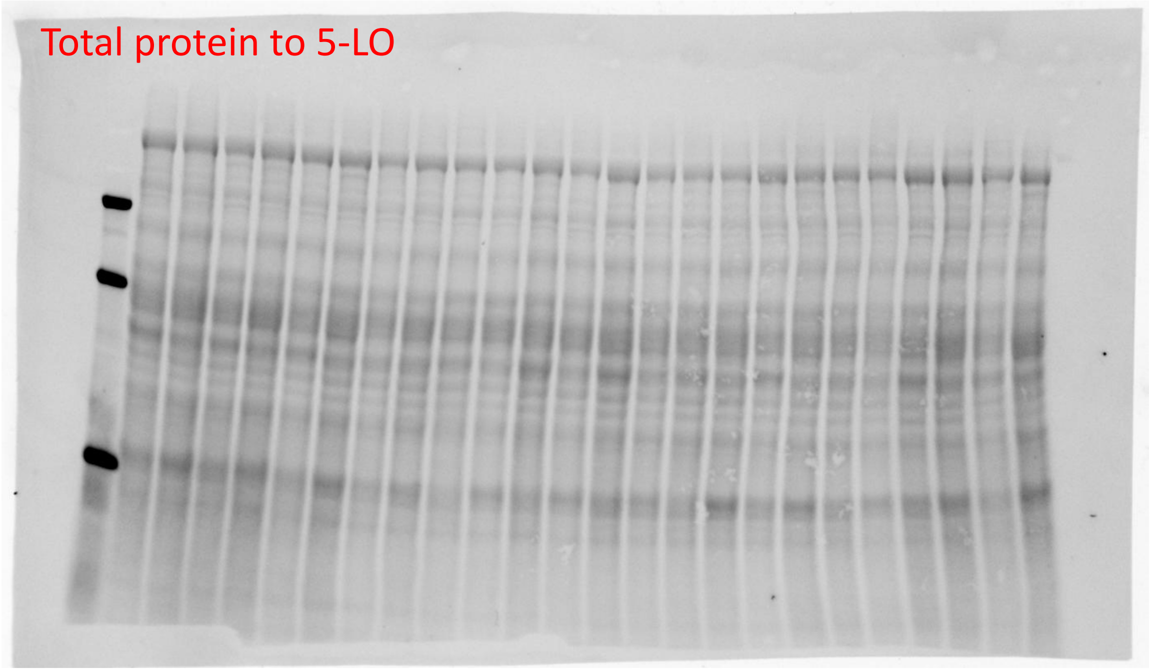
Total protein to 5-LO

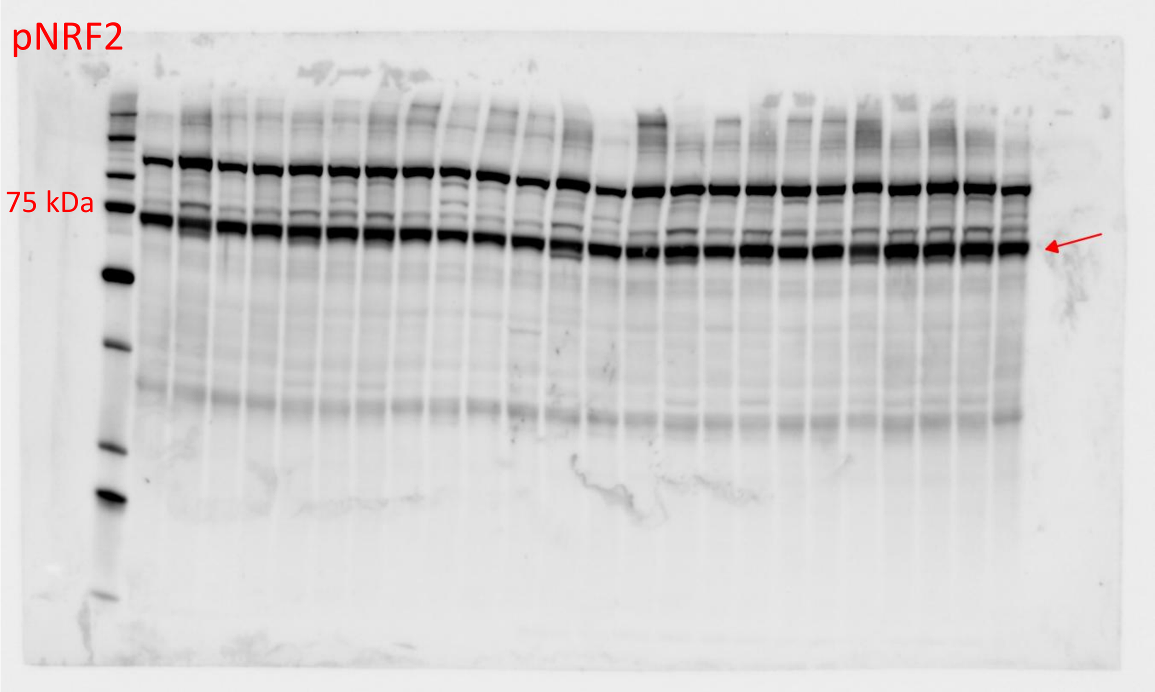
pNRF2
75 kDa

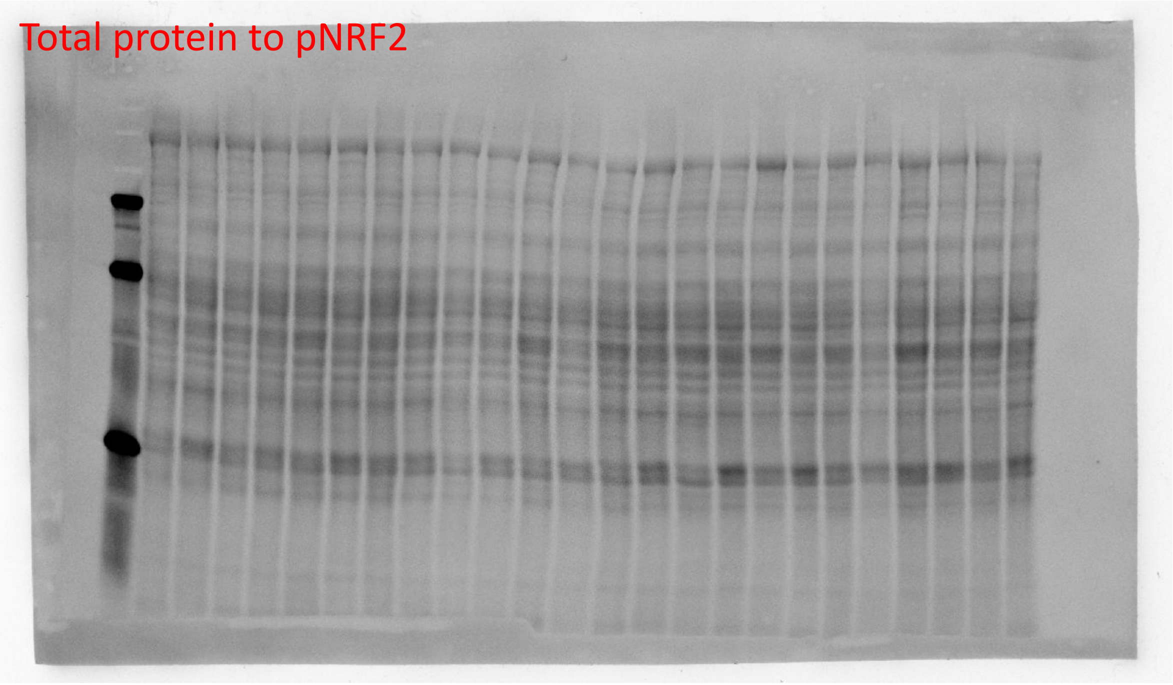
Total protein to pNRF2
